# Variations in water use strategies of *Tamarix ramosissima* at coppice dunes along a precipitation gradient in desert regions of northwest China

**DOI:** 10.3389/fpls.2024.1408943

**Published:** 2024-07-31

**Authors:** Yanqin Xu, Hui Zhao, Binqian Zhou, Zhengwu Dong, Guangying Li, Shengyu Li

**Affiliations:** ^1^ School of Life Sciences, Xinjiang Normal University, Urumqi, China; ^2^ Xinjiang Key Laboratory of Special Species Conservation and Regulatory Biology, School of Life Sciences, Xinjiang Normal University, Urumqi, China; ^3^ Xinjiang Institute of Ecology and Geography, Chinese Academy of Sciences, Urumqi, China

**Keywords:** precipitation gradient, *Tamarix ramosissima*, water sources, stable isotopes, coppice dune

## Abstract

**Introduction:**

The precipitation pattern has changed significantly in arid desert areas, yet it is not clear how the water use strategies of Tamarix ramosissima Ledeb. on coppice dunes along a natural precipitation gradient are affected.

**Methods:**

In this study, the hydrogen and oxygen isotope compositions of xylem water, soil water, precipitation, and groundwater were measured by stable isotope techniques in Huocheng, Mosuowan, and Tazhong. Additionally, the water use strategies of natural precipitation gradient were investigated in conjunction with the MixSIAR model.

**Results:**

The results indicated that the water sources of T. ramosissima exhibited significant variation from semi-arid to hyper-arid areas. In semi-arid areas, T. ramosissima mainly absorbed shallow, shallow-middle, and middle soil water; however, T. ramosissima shifted its primary water sources to middle and deep soil water in arid areas. In hyper-arid areas, it mainly utilized deep soil water and groundwater. In contrast, the water source contribution rate of T. ramosissima exhibited relative uniformity across each layer in an arid area. Notably, in hyper-arid areas, the proportion of groundwater by T. ramosissima was significantly high, reaching 60.2%. This is due to the relatively shallow groundwater supplementing the deep soil water content in the area. In conclusion, the proportion of shallow soil water decreased by 14.7% for T. ramosissima from semi-arid to hyper-arid areas, illustrating the occurrence of a gradual shift in potential water sources utilized by T. ramosissima from shallow to deep soil water and groundwater.

**Discussion:**

Therefore, T. ramosissima on coppice dunes shows flexible water use strategies in relation to precipitation and groundwater, reflecting its strong environmental adaptability. The findings hold significant implications for the conservation of water resources and vegetation restoration in arid areas.

## 1 Introduction

In arid desert areas, the growth of vegetation can effectively prevent desertification and promote the restoration of local habitats (Wang et al., 2008; Zhou et al., 2023). However, plant growth is subject to a variety of challenges, with water playing a crucial role in influencing plant development and overall survival (Su et al., 2020; Ma et al., 2021; Zhang et al., 2022). In situations where a plant experiences water scarcity, the symptoms of wilting and decline can be exacerbated (Zhang et al., 2018). The water use strategies of plants are predominantly influenced by factors such as precipitation, groundwater, and soil water availability (Wang et al., 2017; Gow et al., 2018). When groundwater is unavailable for plant use, precipitation becomes a crucial environmental factor influencing the growth and distribution of plants (Cheng et al., 2006; Zhao et al., 2021). Precipitation influences the soil water content (SWC), which in turn impacts the activity of root water uptake (Fan et al., 2017; Liu et al., 2022b). Precipitation patterns have changed significantly in the arid desert areas of northwest China (Luo et al., 2018), within which the frequency of extreme precipitation events can directly influence the development of plants (Peng and Zhou, 2017; Shi et al., 2020; Wang et al., 2021; He et al., 2022; Yang et al., 2023), prompting them to adjust their water use strategies (Nicotra et al., 2010; Grossiord et al., 2017). For example, in the Loess Plateau region, Zhao et al. (2020b) found that *Robinia pseudoacacia* L. mainly utilizes deep soil water in the dry season, and that the proportion of plant uptake of shallow and medium soil water gradually increases with increasing precipitation. In semi-arid areas, the main water source for riparian plants after experiencing precipitation is shallow soil water, while deep soil water and groundwater are the main water sources in the dry season (Wang et al., 2019; Wu et al., 2019). Despite the ability of desert plants to endure arid conditions, the mechanisms through which they optimize their utilization of scarce water resources warrant additional investigation. In addition, the short-term enrichment of water resources induced by each precipitation event plays a pivotal role in the growth of desert plants, even though precipitation is rare in desert areas (Yang et al., 2015a; She et al., 2016). Consequently, it is essential to investigate the water uptake patterns of desert plants, particularly how they adjust their water use strategies along a precipitation gradient. Such knowledge is crucial for evaluating the adaptability of vegetation restoration and protection in desert areas.

Stable isotopes of hydrogen and oxygen (δD and δ^18^O) exhibit several properties that are scientifically advantageous, such as minimal damage to plants, high sensitivity, and excellent accuracy. As a result, they are commonly used to evaluate and track plant water sources and to study water use strategies in arid and semiarid environments (Rothfuss and Javaux, 2017; Sprenger et al., 2018). Most terrestrial plants have a low concentration of isotopes during plant water uptake prior to transpiration flux (Ellsworth and Williams, 2007; Rothfuss and Javaux, 2017). Therefore, by comparing the isotopic values of xylem water with those derived from potential water sources, such as soil water and groundwater, we can reveal the sources of water for plants (Geris et al., 2017; Barbeta et al., 2019). Existing studies using isotope technology have demonstrated that desert shrubs can use shallow and middle soil water when the surface soil water content is high. However, they switch to utilizing deep soil water and groundwater during periods of severe drought in the dry season (Ma et al., 2021; Pei et al., 2024). In addition, *Tamarix ramosissima* Ledeb. relies more on shallow soil water in floodplain habitats with higher SWC compared to hillsides with low SWC (Su et al., 2020). This suggests that *T. ramosissima* has the ability to adjust its water consumption strategy in response to changes in SWC. The study also found that *T. ramosissima* mainly utilizes shallow and mid-depth soil water in spring, which is replenished by snowmelt water, while deep soil water and groundwater are mainly utilized in summer and fall in the shallow water table of the desert plain area (Tiemuerbieke et al., 2018). It is clear that the utilization of water by plants can be flexibly shifted under various moisture conditions. Therefore, the use of stable isotope technology can reveal the water sources of plants in desert areas, aiding in the comprehension of the water use strategies of desert plants along a natural precipitation gradient.

*Tamarix ramosissima* is a typical deep-rooted desert shrub that is widely distributed in the arid desert areas of Xinjiang (Su et al., 2020). It is one of the key species in the arid desert ecosystem and plays a vital role in resisting wind and sand and maintaining the stability of the desert ecosystem (Yang et al., 2014; Li et al., 2021). Thus far, most research on the water use strategies of *T. ramosissima* has been based on seasonal dynamics and changes in groundwater levels (Wu et al., 2016; Su et al., 2020; Li et al., 2021; Liu et al., 2022a). However, in the arid desert areas of Xinjiang, Northwest China, precipitation patterns have experienced significant changes due to global warming (Ning et al., 2021), and such changes may influence the water use strategies of desert plants in this area. In spite of this, uncertainty remains in our understanding of the water use strategies of *T. ramosissima* at coppice dunes along natural precipitation gradients. To address this knowledge gap, we used stable isotope technology to determine the δD and δ^18^O values of plant xylem water, soil water, and groundwater from *T. ramosissima* at various coppice dunes sites. Specifically, we sought to answer the following questions: (i) Are there differences in the water sources of *T. ramosissima* at coppice dunes along a natural precipitation gradient? (ii) Do changes in precipitation influence the water use strategies of *T. ramosissima* at coppice dunes? (iii) What are the primary factors that influence the water use strategies of *T. ramosissima* at coppice dunes? The overarching aim of answering these questions was to elucidate the water use strategies of desert plants along a natural precipitation gradient and thus provide a scientific basis for better water resource management and ecological restoration in arid desert areas.

## 2 Materials and methods

### 2.1 Study sites

**Figure 1:**
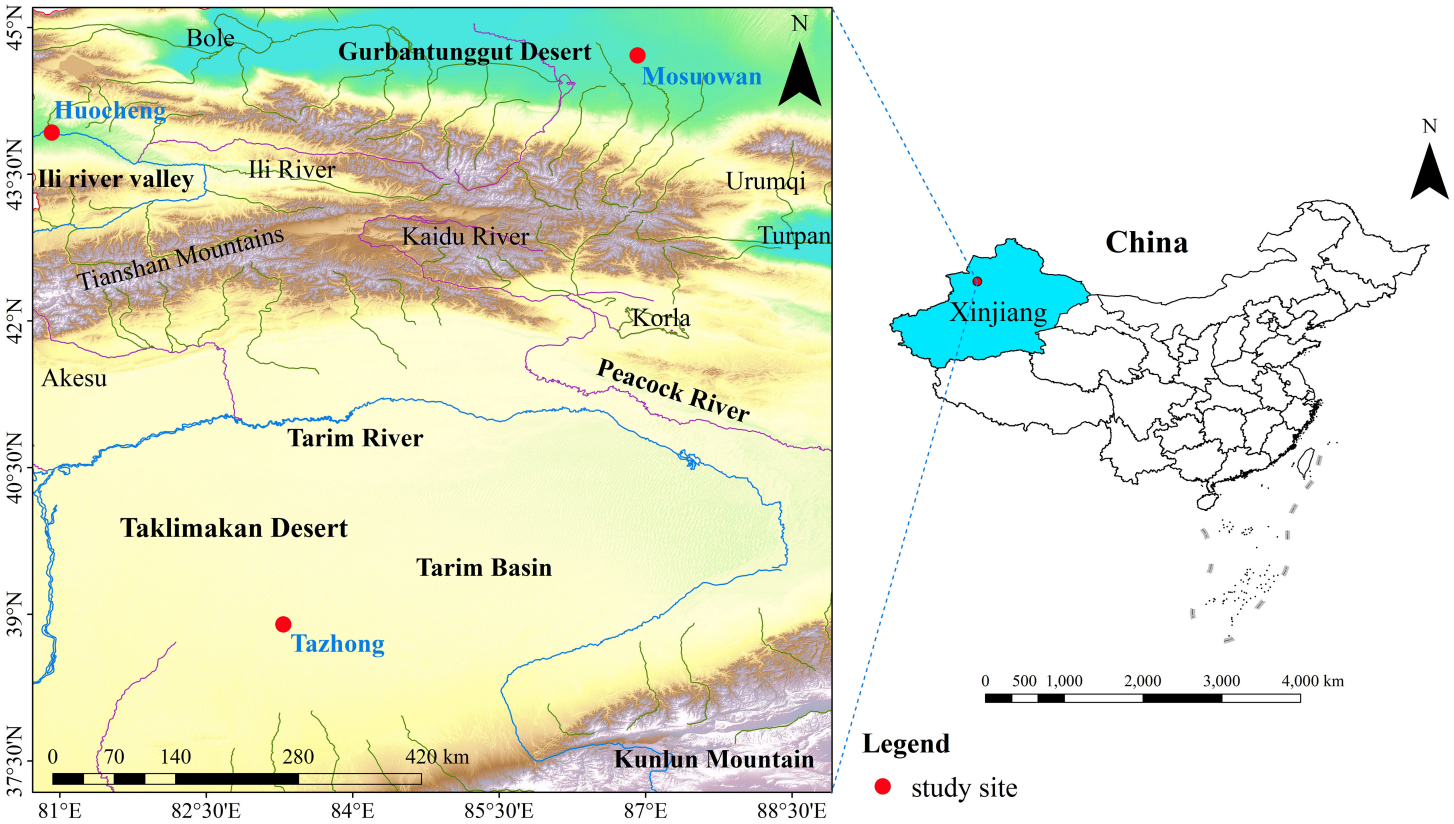
Location of the study sites (red dots) in the desert area of Northwest China.

The study area is located in the Xinjiang Uygur Autonomous Region of China. We selected three representative sites from the north to the south of Xinjiang along a natural precipitation gradient—namely, Huocheng, Mosuowan, and Tazhong (**Figure 1**; **Supplementary Table S1**). Along this gradient, the annual mean precipitation diminishes from 218 mm to 24 mm. Based on the natural precipitation gradient, we categorized Huocheng, Mosuowan, and Tazhong into semi-arid, arid, and hyper-arid areas, respectively. Additionally, all three areas have typically *T. ramosissima* coppice dunes.

Huocheng is located in the Yili Valley area, where the annual average precipitation is 200–500 mm. Precipitation predominantly occurs from March to August, representing 71% of the total annual precipitation (**Supplementary Figure S1**). The annual average evaporation ranges from 1260 to 1900 mm and the annual average temperature from 2.6°C to 9.2°C, thus meaning the site is classified as having a temperate and semi-arid continental climate (Zhao et al., 2019). The groundwater depth is 10–20 m (Huo et al., 2020), and the vegetation in the area is relatively diverse, encompassing trees, shrubs, and herbaceous plants such as *Haloxylon ammodendron* (C. A. Mey.) Bunge, *Caragana sinica* (Buc’hoz) Rehd., and *Halimodendron halodendron* (Pall.) Voss. These species are widely distributed throughout the area, and the total vegetation coverage in this region ranges from 35% to 40% (Yang et al., 2010).

Mosuowan is located on the southwest edge of the Gurbantunggut Desert, where the climate is classified as typical temperate arid desert. The annual average precipitation is approximately 70–180 mm, predominantly occurring from March through October and accounting for 79% of the total annual precipitation (**Supplementary Figure S1**). The average annual air temperature in this area is 6–10°C, while the annual potential evaporation exceeds 2000 mm (Zhao et al., 2023). The groundwater level between the southern edge of the Gurbantunggut Desert and the internal dunes exceeds 16 m (Xu et al., 2022). The soils of the coppice dunes in this area are predominantly composed of 4% clay, 77% silt, and 19% sand (**Supplementary Figure S2**). The vegetation mainly consists of shrubs, trees, and various short-lived herbaceous plants, and the vegetation coverage ranges from 15% to 30% (Yang et al., 2015b). The main dominant species are *H. ammodendron*, *Haloxylon persicum* Bge. ex Boiss. et Buhse., and *T. ramosissima* (Li et al., 2007).

Tazhong is located in the hinterland of the Taklimakan Desert and has a typical extreme arid continental climate. The average annual precipitation is 24–65 mm, which mainly occurs from May to August, making up 73% of the total annual precipitation (**Supplementary Figure S1**). The average annual temperature in this area is 12.4°C, while the average annual potential evaporation is 3,639 mm (Zhang et al., 2016). The groundwater depth is 5–6 m (Dong et al., 2020). The soils of the coppice dunes in this area are predominantly composed of 2% clay, 59% silt, and 39% sand (**Supplementary Figure S2**). The vegetation of the Taklimakan Desert exhibits notably low coverage, limited species diversity, and a simplistic community structure. The main dominant species include *Populus euphratica* Oliv., *T. taklamakanensis* M. T. Liu, and *H. ammodendron* (Li et al., 2015).

### 2.2 Experiment design and sampling

In this study, we chose to collect samples from Huocheng, Mosuowan, and Tazhong, because they are three representative sites in the arid desert areas of Xinjiang.

A 20 × 20m plot was set up in each region, and three typical *T. ramosissima* coppice dunes with good growth and similar morphology were selected in each plot area. The dune heights were all around 2.5 m, and the vegetation on the dunes was well established with few dead branches. According to the growth conditions of local *T. ramosissima*, we collected plant, soil, and groundwater samples from the Mosuowan, Huocheng, and Tazhong sites on 3, 12, and 17 July 2021. These samples were collected on the third day after precipitation, with precipitation amounts of 4.39mm, 2.89mm, and 0.82mm for semi-arid, arid, and hyper-arid areas, respectively. All sampling processes were completed between 10:00 and 11:00 local time to reduce the influence of external conditions such as light intensity on isotope results.

#### 2.2.1 Soil sample collection

We collected soil samples from three randomly selected sample points at the top of each of the selected *T. ramosissima* coppice dunes. Soil samples were taken from 0 cm to 500 cm using a soil auger with a diameter of 5 cm, sampling every 20 cm as a soil layer. For each layer, three replicates of the soil samples were gathered to ensure accuracy and reliability in the subsequent analysis. Soil samples collected from each layer were divided into two parts, one of which was quickly packed into sample bottles sealed with Parafilm to prevent evaporation–distillation, placed in a portable refrigerator, and brought back to the laboratory for freezing (− 20°C) ahead of stable isotope analysis. The other part was packed into an aluminum box and then oven-dried in the laboratory to determine the SWC as follows:


(1) $$\text{SWC}=\frac{M_{1}-M_{2}}{M_{1}-M_{0}}\times 100\%,$$

Where *M*_1_ is the mass of wet soil added to the aluminum box before drying (g), *M*_2_ is the mass of dry soil in the aluminum box after drying (g), and *M*_0_ is the mass of the empty aluminum box (g).

#### 2.2.2 Plant xylem sample collection

Plant samples were collected along with soil samples. On the selected *T. ramosissima* coppice dunes in the three sites, six randomly selected embolized twigs were cut with tree clippers into small sections (roughly 0.5 cm in diameter, with a length of around 3–5 cm). For each sample, three replicates were collected, resulting in a total of 54 twigs. The outer bark and phloem were quickly peeled off, retaining the xylem. Subsequently, the samples were immediately packed into vials and sealed with Parafilm to prevent evaporation–distillation. The samples were then placed in a portable refrigerator and brought back to the laboratory for freezing (− 20°C). These samples were then stored for subsequent extraction of water and stable isotope analysis.

#### 2.2.3 Water sample collection

Groundwater samples were collected at three selected sites, each near a well (approximately 10 km away from each plot), with three replicates for each sample. A total of nine groundwater samples were collected during the sampling period. Before each sampling, the water was drained for 5–10 min. When the well water was cold to the touch, the sample bottles were washed three times with well water. Subsequently, the samples were collected, sealed with Parafilm, and refrigerated at 2°C until isotopic analysis.

#### 2.2.4 Meteorological data collection

The meteorological data for the experiment were sourced from the WheatA wheat germ-agricultural meteorological big data system V1.5.6 (http://www.wheata.cn/). Key meteorological variables included monthly average temperature, monthly average evapotranspiration, and monthly precipitation.

### 2.3 Isotopic analysis

A cryogenic vacuum distillation system (LI-2100Pro, Beijing Lijia United Technology Co. Ltd., Beijing, China) was used to extract plant xylem water and soil water. The samples of plant xylem water, soil water, groundwater, and precipitation were initially filtered using disposable aqueous filters with a pore size of 0.22 μm. Subsequently, they were transferred into 2 ml sample vials that were sealed with Parafilm and refrigerated at a temperature of 2°C until isotopic analysis. Stable hydrogen and oxygen isotopic compositions were determined using a liquid water isotope analyzer (LGR DLI-100, Los Gatos Research, Mountain View, CA, USA). The analytical precision of individual measurements was ± 0.25‰ and ± 1.00‰ for δ^18^O and δD, respectively. In order to eliminate the memory function of the instrument, each sample was measured six times. The stable isotope values can be expressed as:


(2) $$\text{δD}/\text{O}=\left(\frac{{\text{R}}_{\text{sample}}}{{\text{R}}_{\text{standard}}}-1\right)\times 1000\text{ ‰},$$

Where *R*_sample_ and *R*_standard_ are the stable isotope values (D/^1^H and ^18^O/^16^O) of the sample and standard water (standard mean ocean water), respectively. Since the vacuum condensation extraction device will fractionate some methanol and ethanol substances that have similar spectral absorption peaks to water molecules when extracting plant water, the stable isotope values of plant xylem water and soil water may be wrong. In order to reduce the measurement error, the spectral analysis software developed by Los Gatos Research Company (LWIA-SCI) was used to determine the spectral measurement values of the pollution degree of methanol and ethanol substances and to correct the isotopic spectral pollution (West et al., 2011) to obtain more accurate isotope values.

The lc-excess (Landwehr and Coplen, 2006) was used to describe the isotopic offset value between xylem water and its potential water sources of *T. ramosissima* in the three sites, which was calculated as follows:


(3) $$lc-excess=\delta D-a\delta^{18}O-b$$

Here, δD and δ^18^O are the hydrogen and oxygen isotope values of soil water at coppice dunes of different sites; a and b are the slope and intercept of the local meteoric water line (LMWL), respectively.

### 2.4 Classification of water sources

Two methods were used to study the water utilization of *T. ramosissima*. One method directly compared the δ^18^O of xylem water with the δ^18^O of various potential water sources; the potential water sources mainly used by plants are similar to the δ^18^O of xylem water (Ehleringer and Dawson, 1992). The other method used the MixSIAR model calculation to determine the proportion of plants using different potential water sources (Parnell et al., 2013). The MixSIAR model is an installation package used in R. Not only can it analyze multiple potential sources at the same time and run fast, but it can also take into account the differences between isotope values, which can effectively improve the calculation accuracy of the model (Ma and Song, 2016). Potential water sources were divided according to the SWC at different soil-layer depths and the difference in δ^18^O values of soil water. The δ^18^O values of soil water and the SWC in each layer were compared, with multiple repeats, using the least significant difference (LSD) method, with significance tested at the 0.05 level, merging similar adjacent soil layers. The whole soil profile (0–500 cm) was divided into four potential water sources (soil water of 0–80 cm, 80–180 cm, 180–420 cm, and 420–500 cm). The four potential water sources were identified as follows:

(1) Shallow soil layer (0–80 cm): The δ^18^O of soil water is notably influenced by climatic factors such as precipitation, temperature, and evaporation, exhibiting the most significant fluctuations.
(2) Shallow-middle soil layer (80–180 cm): Although the δ^18^O of soil water exhibits significant fluctuations, these variations are relatively gentle when compared to those in the surface layer.
(3) Middle soil layer (180–420 cm): Compared with the above two soil layers, the changes in SWC and isotope ratios are relatively low.
(4) Deep soil layer (420–500 cm): Since it is less affected by the external environment, the δ^18^O of soil water exhibits a consistent trend with increasing soil depth.

### 2.5 Data analysis

The contributions of potential water sources to the *T. ramosissima* were evaluated using the MixSIAR package in R. The correlation between the proportion of water absorption across the range of soil layers and various environmental factors was examined with the corrplot package in R. One-way analysis of variance (ANOVA) was utilized, followed by a *post-hoc* Turkey test (*p* < 0.05), to identify significant differences in the isotope ratios across various water sources in all sites. The relationship between δD and δ^18^O values in soil water was analyzed using a common linear regression model. All figures were drawn in Origin 2021.

## 3 Results

### 3.1 Variations in SWC and stable isotope values

**Figure 2:**
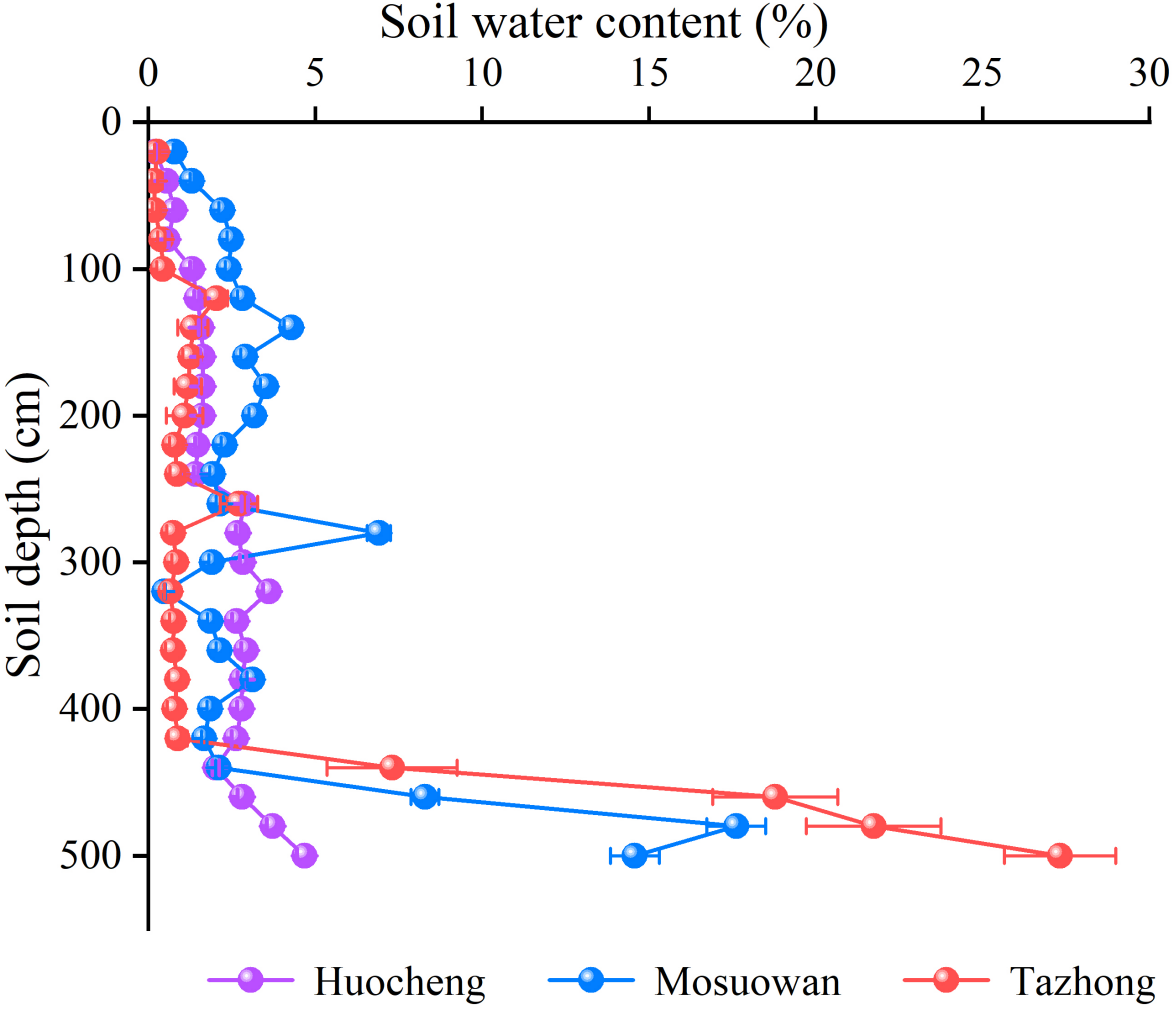
Soil water content of *T. ramosissima* at coppice dunes at all sites. Bars represent standard error bars.

The SWC was observed to be generally low at all sites, but there were significant differences between them (**Figure 2**). The mean values of SWC in arid areas were significantly higher than those in semi-arid and hyper-arid areas (*p* < 0.05), and the same trend was observed for shallow and shallow-middle SWC in arid areas. The SWC in the middle layer followed the order semi-arid (2.52%) > arid (2.45%) > hyper-arid (0.97%), while the deep SWC followed the order hyper-arid (18.78%) > arid (10.64%) > semi-arid (3.30%) (Equation 1). As soil depth increased, there was an increasing trend in SWC across all sites. Particularly noteworthy was the significantly lower SWC in the 0–420 cm soil layer compared to the 420–500cm layer in arid and hyper-arid areas. Additionally, in a semi-arid area, the SWC in the 0–180 cm layer was significantly lower than that in the 180–500 cm layer (*p* < 0.05). The SWC of *T. ramosissima* coppice dunes in semi-arid, arid, and hyper-arid areas exhibited a primary peak at 240–260 cm (2.88% ± 0.09%), 260–280 cm (6.90% ± 0.34%), and 240–260 cm (2.70% ± 0.56%), respectively. The SWC increased significantly with depth in the range of 420–500 cm of soil layer in all sites (*p* < 0.05).

**Figure 3:**
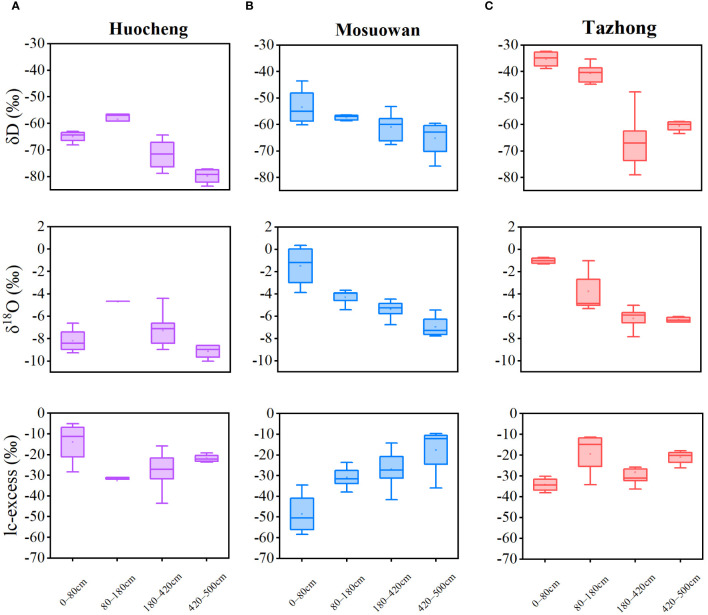
Variation characteristics of soil water isotopes and lc-excess at coppice dunes from semi-arid **(A)**, arid **(B)** and hyper-arid **(C)** areas. Bars represent standard error bars.

The δD and δ^18^O values of soil water at coppice dunes exhibited significant variations across different natural precipitation gradients (**Figure 3**). The average order of δD and δ^18^O was as follows: hyper-arid > arid > semi-arid. The ranges of δD values of soil water in semi-arid, arid, and hyper-arid areas were − 83.57‰ to − 56.47‰, − 69.02‰ to − 43.04‰, and − 79.02‰ to − 32.34‰, respectively; while the ranges of δ^18^O values were − 10.02‰ to − 4.41‰, − 7.12‰ to 2.96‰, and − 7.79‰ to − 0.87‰, respectively (Equation 2). The δD and δ^18^O values exhibited comparable trends across both the arid and hyper-arid areas. Specifically, the average δD and δ^18^O values of soil water in each layer exhibited a decreasing trend from the shallow to deep layer in arid and hyper-arid areas, with the highest values observed at 0−20 cm. However, in semi-arid areas, the mean δD and δ^18^O values of soil water exhibited a significant increase in the shallow-middle layer compared to the shallow layer. Furthermore, both δD and δ^18^O values initially rose and then declined with the depth of the soil layer. As the soil depth increased, the isotopic composition of soil water gradually decreased and stabilized at all sites. The lc-excess values of arid areas were significantly higher than those of semi-arid and hyper-arid areas, suggesting that the evaporation of soil water has the most significant impact in arid areas (Equation 3). The lc-excess value was lowest in the shallow-middle layer of semi-arid areas, while the values were lowest in the shallow layer of arid and hyper-arid areas.

The xylem water and groundwater isotope values of *T. ramosissima* also showed differences from semi-arid to hyper-arid areas (**Table 1**). The δD and δ^18^O values of *T. ramosissima* in both xylem water and groundwater demonstrated a progressive increase from semi-arid to hyper-arid areas, with the δD and δ^18^O values in hyper-arid areas being significantly higher than those in other areas. In semi-arid areas, the δD values of xylem water and groundwater of *T. ramosissima* were notably lower compared to those in other areas (*p* < 0.05). However, in hyper-arid areas, the δ^18^O value of groundwater was significantly higher than that in other areas (*p* < 0.05). There was no significant difference in the δ^18^O of xylem water at all sites (*p* > 0.05).

**Table 1:** Stable isotope values of plant xylem water and groundwater at all sites.

Site	Xylem water		Groundwater	
	δD (‰)	δ^18^O (‰)	δD (‰)	δ^18^O (‰)
Huocheng	− 66.91 ± 1.69c	− 6.97 ± 0.37a	− 84.09 ± 0.15c	− 12.39 ± 0.27b
Mosuowan	− 61.80 ± 3.73b	− 6.74 ± 1.02a	− 72.52 ± 1.92b	− 11.71 ± 0.34b
Tazhong	− 56.65 ± 1.55a	− 6.42 ± 0.33a	− 57.33 ± 3.46a	− 8.06 ± 0.69a

The values in the table represent the mean ± standard deviation; different lowercase letters indicate significant differences in δD or δ^18^O between different study sites.

### 3.2 Relationships between soil water, groundwater, and xylem water isotopes

**Figure 4:**
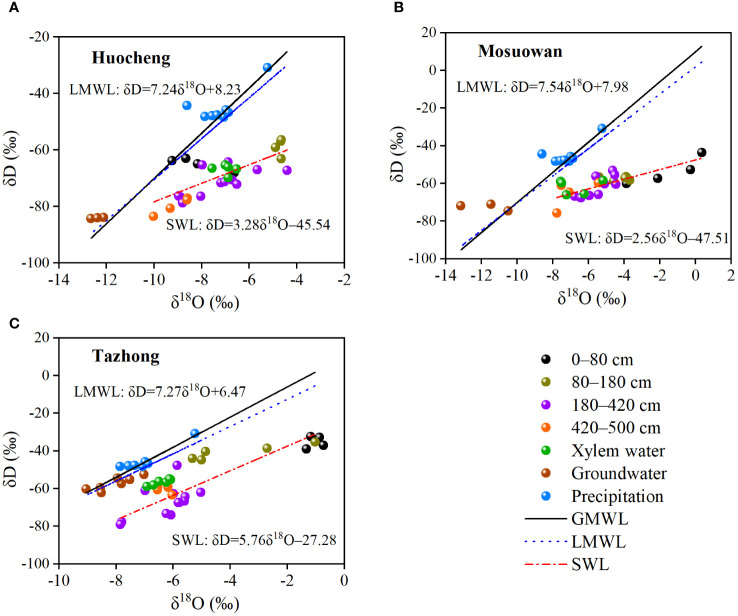
Relationship between δD and δ^18^O in different water bodies of coppice dunes in semi-arid **(A)**, arid **(B)**, and hyper-arid **(C)** areas. SWL, soil water line in three sites (Huocheng: δD = 3.28^18^O − 45.45; Mosuowan: δD = 2.56^18^O − 47.51; Tazhong: δD = 5.76^18^O − 27.28); LMWL, local meteoric water line (Tazhong: δD = 7.27δ^18^O + 6.47 (Zhou et al., 2017); Mosuowan: δD = 7.54δ^18^O + 7.98 (Tiemuerbieke et al., 2018); Huocheng: δD = 7.24δ^18^O + 8.23 (Feng and Yang, 2022)); GMWL, global meteoric water line (δD = 10 + 8δ^18^O (Craig, 1961)).

There was a linear relationship between δD and δ^18^O in precipitation, soil water, xylem water, and groundwater at three sites (**Figure 4**). In semi-arid, arid, and hyper-arid areas, their soil water line (SWL) slopes and intercepts were observed to be significantly lower than those of both the global meteoric water line (GMWL) (δD = 8δ^18^O + 10) and LMWL, indicating that precipitation and soil water at each site have experienced fractionation due to evaporation. The δD and δ^18^O values of soil water at all sites exhibited a deviation from the LMWL, suggesting that soil water was replenished by precipitation and experienced evaporative fractionation effects at each site. In addition, the composition of hydrogen and oxygen isotopes in soil water gradually approached the LMWL as the soil depth increased, indicating a gradual decrease in soil evaporation intensity from the surface layer to the deep layer. The SWL fitted by the linear regression of δD and δ^18^O in hyper-arid areas was steeper compared with that in semi-arid and arid areas. The isotopes of xylem water at the three sites were plotted in soil water intervals at different levels, suggesting that precipitation is not the direct water source for *T. ramosissima* at the various sites. Instead, the soil water from different soil layers serves as the main water source for *T. ramosissima*.

**Figure 5:**
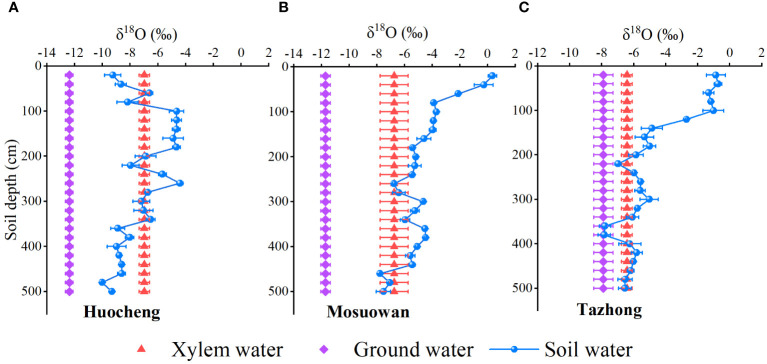
Comparison of δ^18^O values of soil water, groundwater, and xylem water at coppice dunes from semi-arid **(A)**, arid **(B)** and hyper-arid **(C)** areas. Bars represent standard error bars.

Significant variations were observed in the water-absorbing layer position of *T. ramosissim*a transitioning from semi-arid to hyper-arid areas (**Figure 5**). In the semi-arid area, the δ^18^O value of the xylem water of *T. ramosissima* was relatively close to the δ^18^O values of the soil water in the 40–60cm, 80–160cm, 180–220cm, and 260–340cm layers. Thus, we speculate that *T. ramosissima* mainly absorbs shallow, shallow-middle, and middle layers of soil water in arid areas. In the arid area, the δ^18^O value of the xylem water of *T. ramosissima* was close to the δ^18^O values of soil water at 240–280 cm and 440–500 cm. This suggests that *T. ramosissima* mainly utilizes the middle and deep layers of soil water as its primary water source in arid areas. In the hyper-arid area, the δ^18^O value of the xylem water of *T. ramosissim* was close to that of the groundwater and the soil water at 180–240 cm, 300–340 cm, and 400–500 cm, suggesting that *T. ramosissima* may use the middle and deep soil water as well as groundwater as its main water source. Furthermore, the δ^18^O values of soil water at 340–380 cm coincided with those of groundwater in arid areas, indicating that soil water may be replenished by groundwater. From the semi-arid to the hyper-arid areas, the δ^18^O values of xylem water (semi-arid: − 6.88‰; arid: − 6.75‰; hyper-arid: − 6.42‰) exhibited similarities to those of shallow (0–80 cm), middle (180–420 cm), and deep (420–500 cm) soil water, suggesting that *T. ramosissim* gradually utilizes deep soil water as precipitation decreases.

### 3.3 Quantitative analysis of vegetation utilization of various water sources

**Figure 6:**
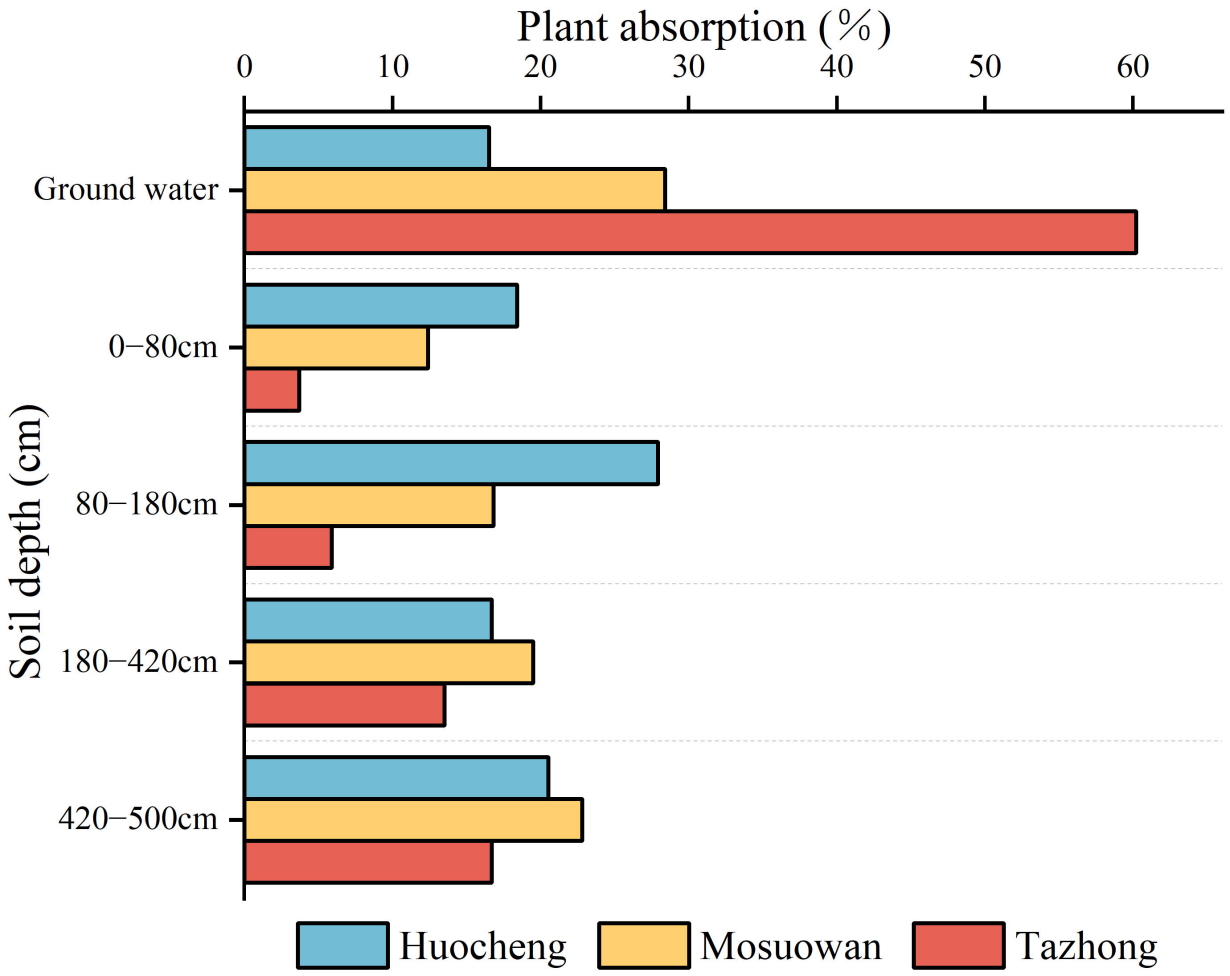
The utilization ratio of potential water sources by *T. ramosissima* at coppice dunes at all sites.

The MixSIAR model was used to quantify the relative contributions of different potential water sources to *T. ramosissim*a in semi-arid, arid, and hyper-arid areas (**Figure 6**). There were differences in water use strategies among the three sites during the study period. In the semi-arid area, the proportions of *T. ramosissima* utilized different soil depths (0–80 cm, 80–180 cm, 180–420 cm, and 420–500 cm) and groundwater were 18.4%, 27.9%, 16.7%, 20.5%, and 16.5%, respectively, suggesting that the plant mainly utilizes shallow, shallow-middle, and deep soil water. In the arid area, the proportions of *T. ramosissima* utilized different soil depths and groundwater was 12.4% (0–80 cm), 16.8% (80–180 cm), 19.5% (180–420 cm), 22.8% (420–500 cm), and 28.4% (groundwater), indicating that the plant predominantly relies on water sources from the middle and deep soil layers as well as groundwater. In the hyper-arid area, the proportions of *T. ramosissima* utilized different soil depths: 3.7% (0–80 cm), 5.9% (80–180 cm), 13.5% (180–420 cm), and 16.7% (420–500 cm). Furthermore, groundwater was a significant contributor to its water source at 60.2%. This shows that the plants in hyper-arid areas mainly utilized deep soil water and groundwater. In semi-arid areas, *T. ramosissima* had the highest utilization rate of soil water, reaching 83.5%. On the contrary, in hyper-arid areas, *T. ramosissima* had the lowest utilization rate of soil water, at 39.8%. The utilization rate of soil water by *T.ramosissima* exhibited a significant increase from hyper-arid to semi-arid areas, with the contribution rate of soil water rising by 43.7%.

### 3.4 Environmental factors affecting water absorption by *T. ramosissima*

**Figure 7:**
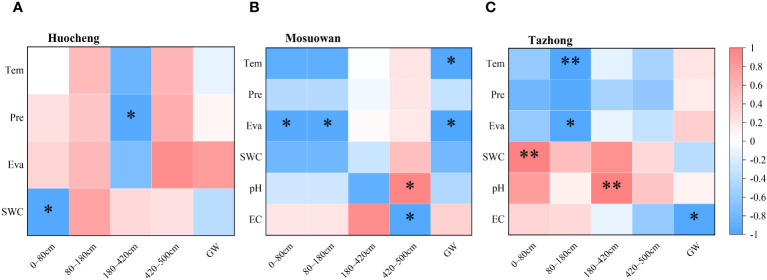
Correlation between environmental factors and the water source utilization efficiency of *T. ramosissima* in semi-arid **(A)**, arid **(B)**, and hyper-arid **(C)** areas. Tem, temperature; Pre, precipitation; Eva, evapotranspiration; SWC, soil water content; EC, electrical conductivity. ^*^ *p* < 0.05; ^**^ *p* < 0.01.

Pearson’s correlation analysis was employed to investigate the correlation between environmental factors and the water absorption fraction of *T. ramosissima* at coppice dunes (**Figure 7**). In semi-arid areas, a significant negative correlation existed between precipitation (Pre) and the water absorption ratio of the 180–420cm soil layer. Similarly, there was a significant negative correlation between SWC and the water absorption ratio of the 0–80cm soil layer, suggesting that the SWC will influence the water use of *T. ramosissima* in semi-arid areas. Other environmental factors were not significantly correlated with the water absorption rate of each soil layer. In arid areas, there was a significant negative correlation between temperature (Tem) and the absorption ratio of groundwater. Similarly, a significant negative correlation was observed between evapotranspiration (Eva) and the absorption ratios of soil layers ranging from 0–80 cm to 80–180 cm, as well as groundwater. Furthermore, a significant positive correlation was found between pH and the water absorption ratio within the 420–500cm soil layer, and conversely, a significant negative correlation was noted between electrical conductivity (EC) values and the water absorption ratio within this same soil layer. In the hyper-arid area, an extremely significant negative correlation was found between Tem and the water absorption ratio of the 80–180cm soil layer. Additionally, there was a significant positive correlation between Eva and the water absorption ratio of the same soil layer. A significant correlation was observed between the SWC and the water absorption rate of the 0–80cm layer, suggesting that plants have difficulty utilizing their shallow soil water when the available water in the soil is low, prompting them to rely on deep soil water instead. Furthermore, there was an extremely significant positive correlation between pH and the water absorption ratio of the 180–420cm soil layer. Conversely, a significant negative correlation was observed between EC and the absorption ratio of groundwater.

## 4 Discussion

### 4.1 Change characteristics of SWC and δD and δ^18^O

The SWC plays a pivotal role in determining the water use pattern and growth of vegetation in desert areas (Ganjegunte et al., 2018; Cao et al., 2020). The SWC plays a pivotal role in determining the water use pattern and growth of vegetation in desert areas (Ganjegunte et al., 2018; Cao et al., 2020). In this study, significant variations in SWC were observed across the sites under a natural precipitation gradient. Notably, the SWC was significantly higher in arid areas compared to the other areas (*p* < 0.05) (**Figure 2**). The observed differences may be attributed to the meltwater replenishment of the arid area. Upon snowmelt, the majority is transformed into soil water via vertical infiltration into the coppice dune soil. The sampling period (July) coincides with the local dry season, during which shallow soil water content progressively diminishes due to increased evaporation. In addition, prior to sampling, the area experienced continuous rainfall, accumulating a total of 5.87 mm, which replenished its surface soil moisture (0–20 cm). On the other hand, arid coppice dunes exhibit a higher soil clay content than hyper-arid areas (**Supplementary Figure S2**), contributing to their higher water-holding capacity (Cao et al., 2020; Dong et al., 2020; Liu et al., 2021). Consequently, the SWC in arid areas surpasses that of other areas. In arid desert areas, the influence of precipitation on soil moisture is limited, with larger precipitation events (> 10 mm) having certain replenishment effects on soil water in the > 40cm layer (Li et al., 2013; Wu et al., 2023). As a result, when precipitation is low, the changes in surface SWC are also minor, and the roots within the surface soil become inactive during drought conditions (Kulmatiski et al., 2020). In this study, the SWC of the shallow layer at all three sites was found to be notably low, which prevented the plants from absorbing water efficiently from the shallow layer and forced them to depend on groundwater or deeper soil water (Wang et al., 2019). In a semi-arid area, the SWC of the shallow layer was lower than that of the shallow-middle layer (**Figure 2**). Despite the fact that precipitation in semi-arid areas exceeds that at other areas (**Supplementary Figure S1**), it is not enough to compensate for the substantial water demand for plant growth and intense evaporation rates (Li et al., 2013). Consequently, *T. ramosissima* predominantly absorbs water from the shallow-middle soil (**Figures 5**, **6**). In the arid and hyper-arid areas, meanwhile, monthly average precipitation is notably sparse, leading to soil water being insufficient for effective replenishment, with plants exhibiting adaptive mechanisms to drought conditions by augmenting the utilization of deep soil water. In arid and hyper-arid areas, the SWC of the deep layer were both significantly higher than that of the semi-arid areas, indicating that the deep soil water in these sites is the main source of water (**Figure 6**). Furthermore, the groundwater depth in semi-arid areas is relatively shallow, at approximately 6 m, which serves as a supplementary source of deep soil water (Zhang et al., 2018), so the deep SWC in hyper-arid areas was significantly higher than that in the other areas.

In general, the δD and δ^18^O values of soil water are predominantly influenced by two primary processes: evaporation and infiltration (Ehleringer and Dawson, 1992; Pei et al., 2024). In this study, the mean values of δD and δ^18^O in soil water of hyper-arid areas were found to be higher than those recorded in semi-arid and arid areas (**Figure 3**), suggesting that the intensity of evaporation of soil water in hyper-arid areas surpasses that of the other sites. Furthermore, its low precipitation impeded the ability of *T. ramosissima* to utilize shallow soil water in hyper-arid areas, leading to a mere 3.7% contribution (**Figure 6**). Shallow soil water exhibits isotopic enrichment due to evaporation fractionation, leading to a decrease in SWC and an increase in δD and δ^18^O values (Liu et al., 2018). In contrast, we observed a trend toward lower δ^18^O values in shallow soil water in semi-arid areas (**Figure 3**). Additionally, lc-excess effectively delineates the intensity of soil water evaporation fractionation signals across various soils in arid areas (Qiu et al., 2023). Notably, in semi-arid areas, the shallow soil exhibited the highest lc-excess value (**Figure 3**), suggesting that its evaporation intensity is relatively low. This might be attributable to the inhibition of shallow soil layer isotopic enrichment following precipitation infiltration (Song et al., 2016), which would indicate that precipitation can infiltrate into the soil surface layer to serve as a water source for *T. ramosissima* in semi-arid areas. In arid and hyper-arid areas, the δD and δ^18^O values of shallow-layer soil water displayed significant fluctuations, which gradually stabilized as the soil layer depth increased. This suggests that the δD and δ^18^O values of the surface soil at these sites are significantly influenced by the substantial evaporation intensity. This finding aligns with the observed trend of SWC in this study and is consistent with previous research (Wu et al., 2016; Liu et al., 2018; Pu et al., 2020). Correspondingly, the lc-excess values of soil water also exhibited an increase with the depth of the soil layer. The lower lc-excess values in shallow soils suggest a higher evaporation intensity within these soils (Pei et al., 2023), which further illustrates that the proportion of shallow soil water utilization is lower in arid and hyper-arid areas.

Previous studies have demonstrated that the slope of the SWL is typically lower than that of the LMWL owing to the evaporative enrichment of stable isotopes in soil water (Benettin et al., 2018; Sprenger et al., 2018; Wang et al., 2020). In this study, the slopes of the SWL and LMWL were all found to be lower than those of the GMWL at all sites (**Figure 4**), suggesting that the precipitation and soil water have undergone fractionation as a result of evaporation. The slopes and intercepts of the LMWL at the three sample sites were clearly smaller than those of the GMWL, suggesting that the stable isotope values were affected by secondary evaporation during precipitation. There were differences in the slope and intercept of the SWL at all sites (**Figure 4**), which may be related to differences in evaporation intensity at coppice dunes across different sites (Pei et al., 2023). In this study, the SWL line in hyper-arid areas was steeper than that in arid and semi-arid areas, indicating higher evaporation intensity in hyper-arid areas. The isotopic compositions of xylem water at all sites are plotted in proximity to the soil water within various soil layers, suggesting that soil water at different depths serves as a direct water source for *T. ramosissima*, indeed, similar conclusions have been reached previously (Su et al., 2020). In semi-arid areas, the δD and δ^18^O values of a part of the soil water were distributed around the LMWL, suggesting that precipitation can serve as a supplementary source for its soil water. In hyper-arid areas, the isotopic values of groundwater are distributed near those of soil water, while in semi-arid and arid areas, there is a significant deviation between the isotopic values of groundwater and soil water. This indicates that groundwater can serve as a source to replenish soil water in hyper-arid areas, but this is not the case in other areas. In summary, the soil water content and the composition of soil isotopes were influenced by precipitation and evaporation. Consequently, *T. ramosissima* at each site adjusts its water absorption depth in response to fluctuations in precipitation and SWC. When soil moisture is adequate, *T. ramosissima* primarily utilizes shallow layers of water. However, in instances of water scarcity, it shifts toward deeper soil water or groundwater sources (Fan et al., 2017; Cao et al., 2020; Ma et al., 2021).

### 4.2 Water utilization strategies and influencing factors of *T. ramosissima* at coppice dunes

**Figure 8:**
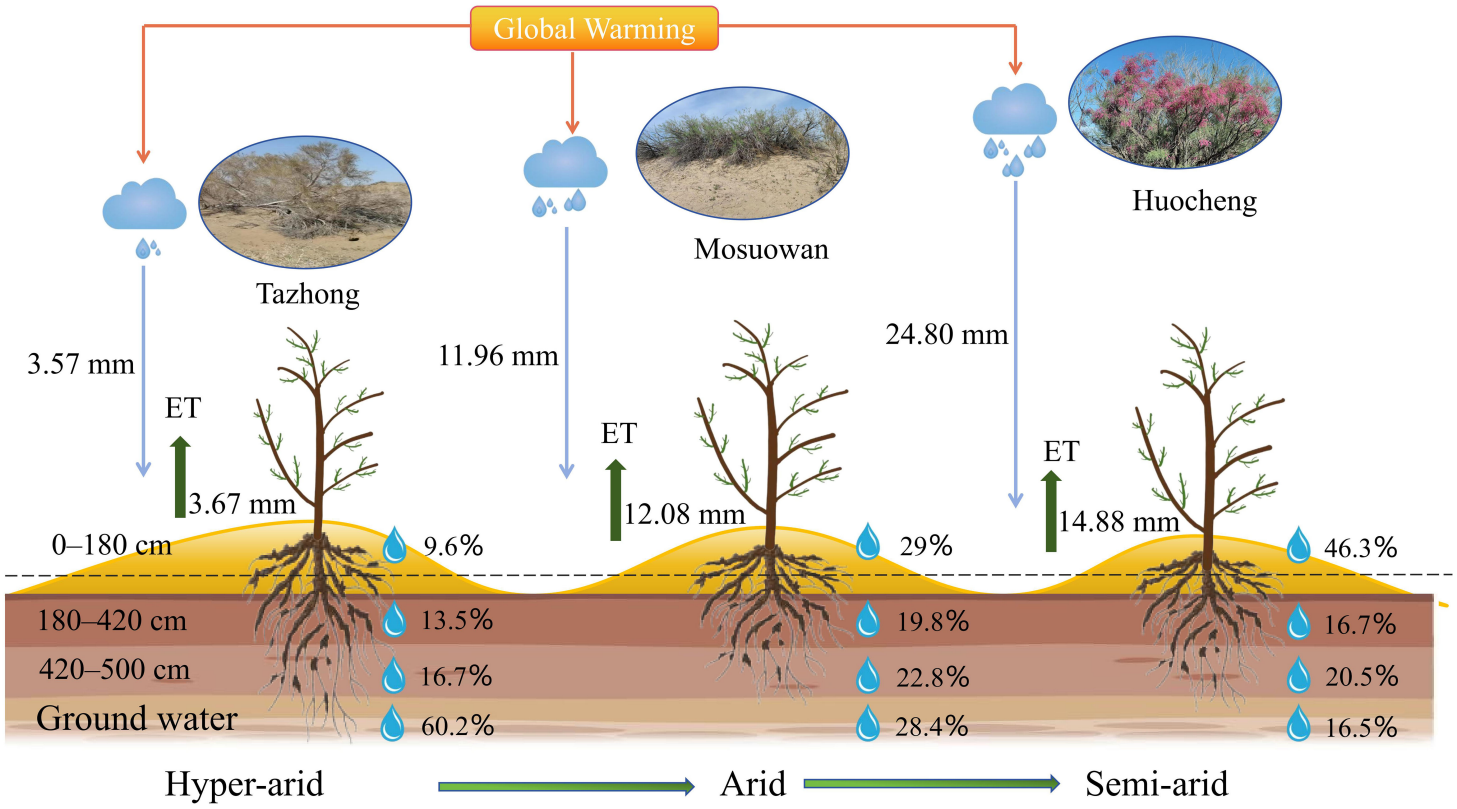
Conceptual model of potential water sources for *T. ramosissima* at coppice dunes along a precipitation gradient. The blue and green arrows represent the average precipitation and evapotranspiration in July from 2020 to 2022, respectively. The percentages illustrate the utilization of soil water at varying layers and groundwater by *T. ramosissima* across different areas.

Water plays a crucial role in facilitating plant growth. Plants exhibit varying water use strategies depending on their respective habitats, with significant variations in water sources, particularly notable in desert areas (Nie et al., 2012). The water use patterns of plants are influenced by factors such as root distribution and water availability. Several studies have demonstrated that precipitation levels of varying magnitudes can affect rates of plant water contribution in different habitats (Loarie et al., 2009; Ma et al., 2021; He et al., 2022). The water use of plants is influenced by the quantity of precipitation, which gradually shifts from the low to the high water soil layer as the amount of precipitation increases (Liu et al., 2016; Zhao et al., 2020a). In this study, *T. ramosissima* was found to be dependent on soil water from the shallow, shallow-middle, and middle layers in semi-arid areas, which may be related to the higher precipitation in this area (**Figure 8**). When precipitation infiltrates the soil, the shallow layers are particularly responsive to changes in SWC, and consequently, plants tend to absorb water from these shallow soil layers during root transport, their aim being to reduce energy expenditure (Hasselquist and Allen, 2009; Wang et al., 2017). However, when conditions in the shallow soil become inadequate for water uptake, plants shift their focus to deeper soil layers (Liu et al., 2018). In the present study, *T. ramosissima* mainly utilized middle and deep soil water in arid areas, with its water use strategy being intimately associated with the SWC, and showing a preference for soil layers with high water content, similar to the results of previous studies (Yang et al., 2015a; Dai et al., 2022). However, in arid areas, the proportion of use in the shallow and shallow-middle layers was smaller, likely due to the lc-excess of the shallow layer being notably lower than that of the middle and deep layers. This suggests that the evaporation rate of shallow soil water is high, prompting plants to adapt to the arid conditions by drawing upon deeper soil water. Consequently, *T. ramosissima* has adapted by utilizing a larger proportion of deep soil water. Although the proportion of *T. ramosissima* using groundwater reached 28.4% in arid areas, the groundwater level was more than 30 m deep, meaning it may be difficult for the roots of *T. ramosissima* to reach the depth of groundwater. In hyper-arid areas, *T. ramosissima* primarily absorbed deep soil water and groundwater. It had a high utilization rate of groundwater, at 60.2%, with a rate of only 3.7% for shallow soil water and 5.9% for shallow-middle soil water. This was due to the precipitation of 0.82 mm (< 5 mm) during the sampling month and the fact that soil situated at depths of more than 10 cm remains largely unaffected by such precipitation (Dai et al., 2020). Moreover, the correlation between precipitation and the water absorption ratio of each soil layer was found to be insignificant in hyper-arid areas (**Figure 7**), suggesting that precipitation has little effect on shallow soil replenishment. In the hyper-arid areas, factors such as low precipitation, high air temperatures, and intense evaporation (**Supplementary Figure S1**) have accelerated the evaporation rate of surface soil moisture, which resulted in the extremely low SWC in shallow layers in the hyper-arid area (**Figure 2**). When the water demand of plants increases, *T. ramosissima* adjusts its water utilization pattern by relying on stable deep soil water and groundwater, using them as the primary water sources for plant growth.

Precipitation is an essential resource for the growth and survival of plants, so variations in precipitation significantly influence plant water use strategies and diversity (Loarie et al., 2009; He et al., 2022). From the semi-arid area to the hyper-arid area, the δ^18^O values of xylem water (semi-arid: − 6.88‰; arid: − 6.75‰; hyper-arid: − 6.42‰) exhibited similarities to those of shallow (0−80 cm), middle (180−420 cm), and deep (420−500 cm) soil water, respectively. Moreover, the proportion of shallow soil water utilized by *T. ramosissima* decreased by 14.7% from the semi-arid to hyper-arid areas (**Figure 6**). The potential water sources utilized by *T. ramosissima* gradually shifted from shallow to deep soil water and groundwater, with a decrease in natural precipitation. It was found that *T. ramosissima* tended to choose shallow soil water in the semi-arid area with its relatively high precipitation (**Figure 5A**), with plants then acquiring deep soil water when the shallow soil moisture is insufficient (**Figures 5B, C**). It has been shown that *T. ramosissima* has a dimorphic root system and the ability to switch its water absorption between shallow and deep soil layers (Wang et al., 2019). In arid environments, the root system remains dormant because the surface soil is affected by the dry climate, and *T. ramosissima* relies on deep roots to absorb deep soil moisture in order to ensure survival (Kulmatiski et al., 2020; Ma et al., 2021). These results demonstrate that *T. ramosissima* has regional variations in its water sources, which are strongly influenced by the rainfall patterns of their respective environments. This observation underscores the adaptability of *T. ramosissima* in its water usage patterns, particularly under drought conditions. It demonstrates that *T. ramosissima* has plasticity in utilizing potential water sources, which can maintain the vitality of the root system to the maximum extent and enable plants to obtain more stable water sources.

## 5 Conclusion

This study used stable isotope technology in conjunction with the MixSIAR model to analyze the water sources and water use strategies of *T. ramosissima* at coppice dunes along a natural precipitation gradient. The findings indicate that alterations in precipitation can induce a shift in plant water use patterns, optimizing the utilization of available water resources. Specifically, *T. ramosissima* primarily utilizes shallow, shallow-middle, and middle soil water in semi-arid areas, while it predominantly relies on middle and deep soil water in arid areas. In hyper-arid areas, its main water source transitions to deep-soil water and groundwater. The proportion of shallow soil water decreased by 14.7% for *T. ramosissima* from semi-arid to hyper-arid areas, illustrating the occurrence of a gradual shift in potential water sources utilized by *T. ramosissima* from shallow to deep soil water and groundwater. The water use strategies of *T. ramosissima* were significantly influenced by changes in precipitation and soil water content across all sites. In conclusion, the water use patterns of *T. ramosissima* across diverse sites exhibit a high degree of flexibility along a natural precipitation gradient. Consequently, *T. ramosissima* utilizes a variety of water utilization strategies to modulate its growth characteristics in response to varying precipitation conditions. The primary objective is to maximize the acquisition of water, which reflects its strong adaptability and increases its competitive advantage over limited water resources in the desert. The findings offer a scientific foundation for the management of water resources and the restoration of ecological systems in arid desert areas.

## Data availability statement

The original contributions presented in the study are included in the article/**Supplementary Material**, further inquiries can be directed to the corresponding author/s.

## Author contributions

ZD: Funding acquisition, Methodology, Writing – review & editing, Data curation. YX: Data curation, Methodology, Writing – original draft. HZ: Writing – original draft, Data curation. BZ: Data curation, Writing – review & editing. GL: Investigation, Writing – review & editing. SL: Methodology, Writing – review & editing.

## Supplementary material

Supplementary Figure 1 Average monthly precipitation (mm), average monthly evapotranspiration (mm), and average monthly temperature (|°C) at Huocheng **(A),** Mosuowan **(B)**, and Tazhong **(C)**. These values represent monthly averages for the period 2020–2022.

Supplementary Figure 2 Vertical distribution of soil particle size composition at Tazhong **(A)** and Mosuowan **(B)**. Data from Dong et al. (2020). The soil particle size composition is described as the percentage of clay (<0.002 mm), silt (0.002–0.050 mm), and sand (0.050–0.250mm).
