# Supplementary figures and images for: Variations in water use strategies of Tamarix ramosissima at coppice dunes along a precipitation gradient in desert regions of northwest China
Source: Front Plant Sci. 2024 Jul 31;15:1408943. doi: 10.3389/fpls.2024.1408943 (PMC11325590; doi:10.3389/fpls.2024.1408943)

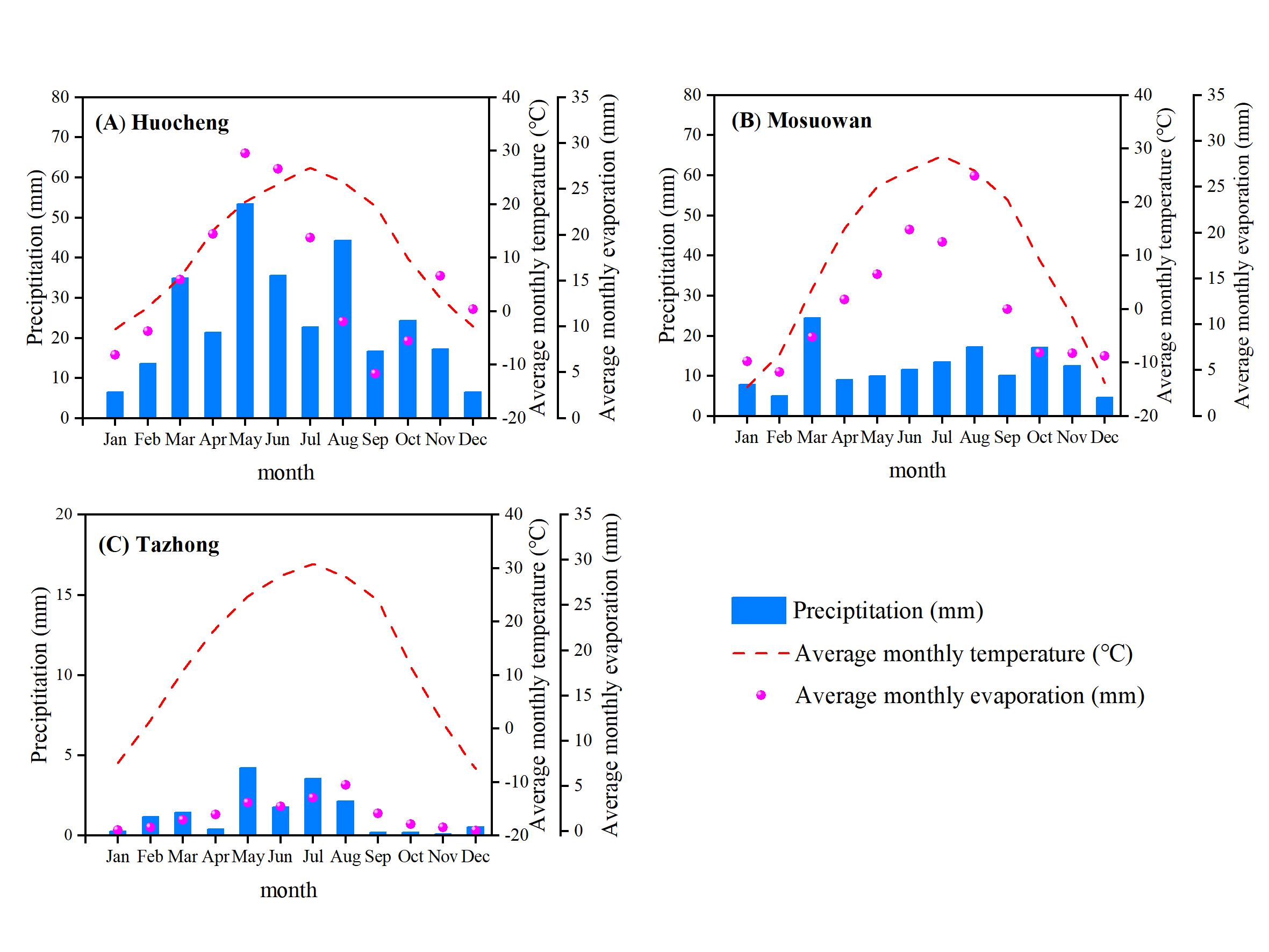

Supplement: Supplementary Figure 1 — Average monthly precipitation (mm), average monthly evapotranspiration (mm), and average monthly temperature (|°C) at Huocheng (A), Mosuowan (B), and Tazhong (C). These values represent monthly averages for the period 2020–2022. [file Image_1.jpeg]

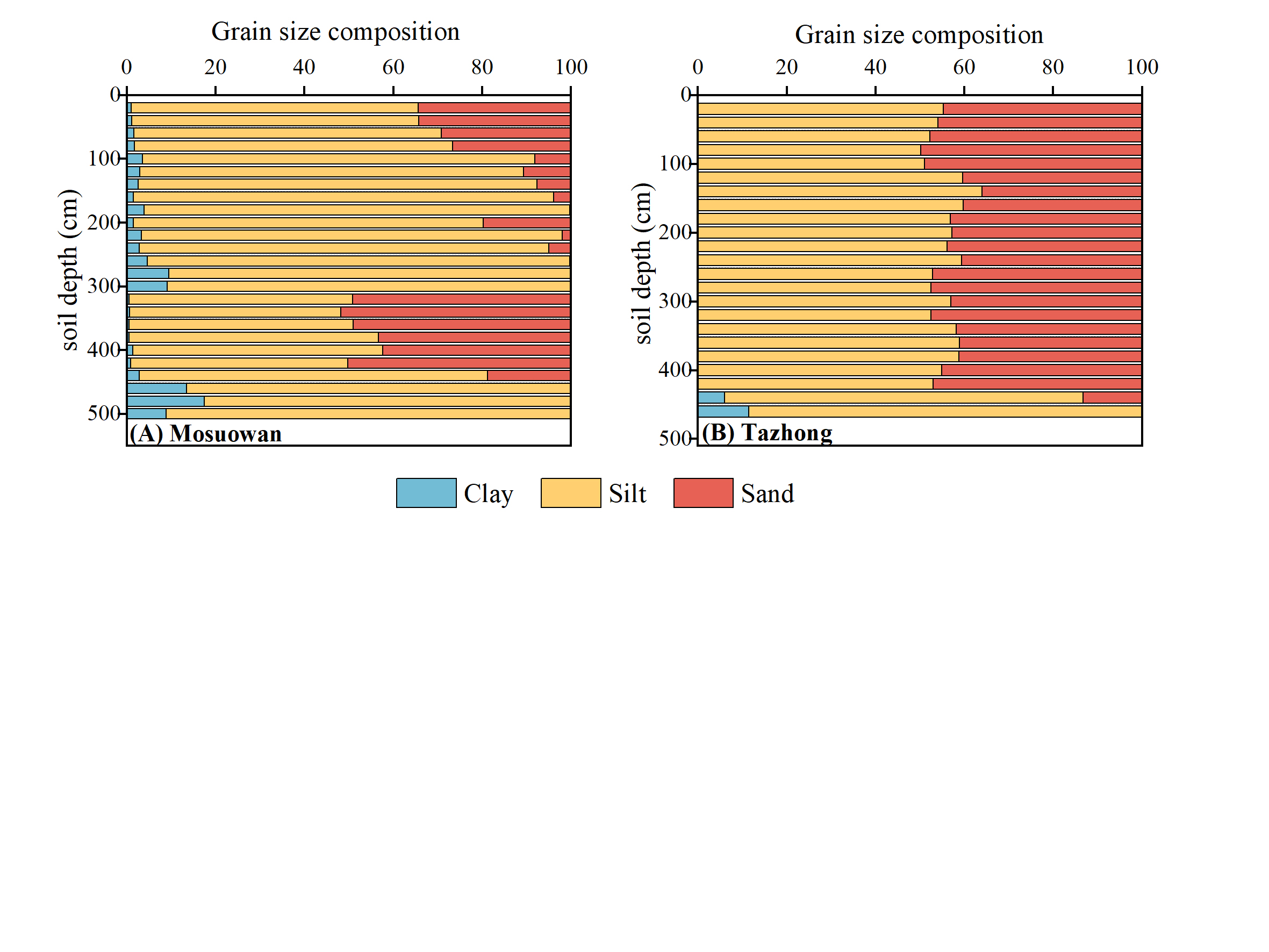

Supplement: Supplementary Figure 2 — Vertical distribution of soil particle size composition at Tazhong (A) and Mosuowan (B). Data from Dong et al. (2020). The soil particle size composition is described as the percentage of clay (<0.002 mm), silt (0.002–0.050 mm), and sand (0.050–0.250mm). [file Image_2.jpeg]
