# Supplementary material for: Variations in water use strategies of Tamarix ramosissima at coppice dunes along a precipitation gradient in desert regions of northwest China
Source: Front Plant Sci. 2024 Jul 31;15:1408943. doi: 10.3389/fpls.2024.1408943 (PMC11325590; doi:10.3389/fpls.2024.1408943)
Supplement: Supplementary file 3 [file Table_1.docx]

Supplementary table 1 Meteorological and habitat data of desert sampling sites in northwest China.

| Site | Desert name | Climate | Height of  scrub dunes | GW |
| --- | --- | --- | --- | --- |
| Huocheng | Ili basin | Semi-arid | 2.5 m | 10–20 m |
| Mosuowan | Gurbantunggut Desert | Arid | 2.5 m | 20–25 m |
| Tazhong | Taklimakan Desert | Extremely arid | 2.5 m | 5–6m |

Notes: GW represents groundwater. The depth of the groundwater table at the meteorological tower is 5–6 m (Dong et al., 2020); the depth of the groundwater table at Mosuowan is 20–25 m (Wu et al., 2019); and the groundwater level at Huocheng is 10–20 m (Huo et al., 2020).
